# Static Range of Motion of the First Metatarsal in the Sagittal and Frontal Planes

**DOI:** 10.3390/jcm7110456

**Published:** 2018-11-21

**Authors:** Sandra Priscila Tavara-Vidalón, Manuel Ángel Monge-Vera, Guillermo Lafuente-Sotillos, Gabriel Domínguez-Maldonado, Pedro V. Munuera-Martínez

**Affiliations:** 1 Department of Podiatry, University of Seville, 41009 Seville, Spain; priscilatavara16@gmail.com (S.P.T.-V.); glafuente@us.es (G.L.-S.); pmunuera@us.es (P.V.M.-M.); 2 Department of Design Engineering, University of Seville, 41011 Seville, Spain; amonge@us.es

**Keywords:** first ray, dorsiflexion, plantarflexion, inversion, eversion

## Abstract

The first metatarsal and medial cuneiform form an important functional unit in the foot, called “first ray”. The first ray normal range of motion (ROM) is difficult to quantify due to the number of joints that are involved. Several methods have previously been proposed. Controversy exists related to normal movement of the first ray frontal plane accompanying that in the sagittal plane. The objective of this study was to investigate the ROM of the first ray in the sagittal and frontal planes in normal feet. Anterior-posterior radiographs were done of the feet of 40 healthy participants with the first ray in a neutral position, maximally dorsiflexed and maximally plantarflexed. They were digitalized and the distance between the tibial malleolus and the intersesamoid crest in the three positions mentioned was measured. The rotation of the first ray in these three positions was measured. A polynomic function that fits a curve describing the movement observed in the first ray was obtained using the least squares method. ROM of the first ray in the sagittal plane was 6.47 (SD 2.59) mm of dorsiflexion and 6.12 (SD 2.55) mm of plantarflexion. ROM in the frontal plane was 2.69 (SD 4.03) degrees of inversion during the dorsiflexion and 2.97 (SD 2.72) degrees during the plantarflexion. A second-degree equation was obtained, which represents the movement of the first ray. Passive dorsiflexion and plantarflexion of the first ray were accompanied by movements in the frontal plane: 0.45 degrees of movement were produced in the frontal plane for each millimeter of displacement in the sagittal plane. These findings might be useful for the future design of instruments for clinically quantifying first ray mobility.

## 1. Introduction

The first ray is a functional unit that is made up of the first metatarsal and the medial cuneiform [1,2,3,4]. First ray mobility (henceforth FRM) is an important component of the normal function of the foot during gait. This mobility has received the attention of many authors over the years and has been related with different pathologies of the foot and the locomotor system [5,6,7,8,9,10,11,12].

The mobility of the joints between the first metatarsal and the medial cuneiform (first tarsometatarsal joint, henceforth first TMTJ), and between this and the navicular (medial cuneonavicular joint, henceforth MCNJ), is produced around a common axis [4,5,6,7,8,9,10,11,12,13]. This axis was first described by Hicks in 1953 [13], having an approximate inclination of 45 degrees with respect to the sagittal and frontal planes and only a slight inclination with respect to the transversal plane. Although the first ray thus has tri-axial mobility, due to the fact that its movement axis is almost parallel to the transversal plane, the movements considered clinically relevant are those that are produced in the sagittal plane and frontal plane [1].

The first ray normal range of motion is difficult to quantify due to the number of joints which participate in it. Roukis and Landsman carried out a review of the literature that revealed the global inconsistency that exists with respect to the study of the FRM [3]. Different methods have been used to quantify this mobility. The manual evaluation is subjective and has a low reproducibility and validity [14]. Some studies have employed more complex equipment, but they only quantify the mobility in the sagittal plane, ignoring the changes which take place in the frontal plane [6,9,10,15,16,17,18]. Other studies are centered only on valuing the movement in the first cuneometatarsal joint, due to the difficulty of precisely measuring the mobility in each of the small joints involved [6,15,16,17,18,19]. Other authors have valued the movement of all these joints in cadavers, although in these studies the in vivo conditions of the foot are not reflected [17,18,19,20].

Discrepancy exists as to the quantification of the normal movement in the sagittal plane and there are few studies about the normal value of movement in the frontal plane of the first ray. Therefore, the purpose of this research was to study the FRM via a radiographic procedure in a sample of feet with normal first rays. In addition, it aimed at obtaining a math equation that describes an average curve of FRM in the sagittal and frontal planes.

## 2. Methods

### 2.1. Participants

The sample of this study was made up of healthy volunteers, adult students, or those accompanying patients who went to the Podiatry Clinical Area of the University of Seville, Spain, who did not have morphological or functional alterations in the first ray. Forty volunteers initially took part. All of the participants gave their written consent before being included in the study. Authorization was obtained from the Podiatry Clinical Area of the University of Seville, and from the Ethical Research Committee of the University Hospitals “Virgen Macarena” and “Virgen del Rocío” of Seville (approval date: September 2016; project code: 1586-N-16).

The inclusion criteria were: adults with normal feet [21], without first ray morphological or functional alterations, and with first ray normal mobility [14]. People who had experienced a traumatism or surgical intervention in the first ray had hallux limitus, hallux rigidus, or hallux abducto-valgus, or had suffered inflammatory, metabolic processes, or degenerative or neuromuscular diseases affecting their feet, were excluded from the study. Those participants in whose radiographs two different examiners could not clearly identify the points to mark were excluded. Participants were recruited between November 2016 and April 2017.

### 2.2. Study Design

A manual examination of the FRM was carried out via the classic clinical maneuver that was described by Root et al. [22]. To include a subject in the study, two explorers, podiatrists having more than 15 years of expertise, had to coincide separately in their valuation of the first ray as “normal” using this maneuver.

To do so the volunteer was placed on the examining table in the dorsal position with his/her ankle relaxed and the subtalar joint in a neutral position. With one hand the explorer held the heads of the second to the fifth metatarsals and with the other held the head of the first metatarsal. In this position, the head of the first metatarsal was moved upward to tis maximum length (dorsiflexion) and after moved downward to its maximum length (plantarflexion). The range of movement was determined by comparing the position of the explorer’s index fingers and the thumbs when making the movements. The neutral position from which the dorsiflexion and the plantarflexion of the metatarsal had to be valued was that in which the head of the first metatarsal was in the same plane as the rest of the metatarsal heads. The subjects who were selected had apparently equal movements of dorsiflexion and plantarflexion. If the two feet were normal, then the choice was made randomly by tossing a coin in the air. If the subject had one normal foot and the other not normal, then the normal foot was selected.

When a participant was included in the study, three radiographs were done using the modified Coleman’s block test, as described by Fritz and Prieskorn, to quantify the first ray maximum dorsalflexion and plantarflexion [15]. In order to know the thickness of the block, the participant was placed standing on a podoscope, and hard polyethylene foam blocks, 2 to 5 mm thick, were progressively placed under the first metatarsal to quantify the maximum dorsalflexion. It was determined that the first metatarsal had reached maximum dorsalflexion when the head of the second metatarsal began to become unweighted. The thickness of the blocks that had to be applied in the Coleman’s test was quantified. The same procedure was followed for maximum plantarflexion, placing the blocks under the lesser metatarsal. It was determined that the first metatarsal was in maximum plantarflexion when the head of the first metatarsal began to lose support.

Three A-P radiographs of the foot were done: one with the first ray in a neutral position, one with the first ray in maximum dorsalflexion, and one with the first ray in maximum plantarflexion, using the blocks, as has been mentioned above. These images were used to quantify the ROM of the first ray in the sagittal and frontal planes. Radiographs were digitalized using an Epson Expression 1680 Pro^®^ scanner (Seiko Epson Corporation, Tokyo, Japan) that can explore images in positive films.

### 2.3. Measurements

**Figure 1:**
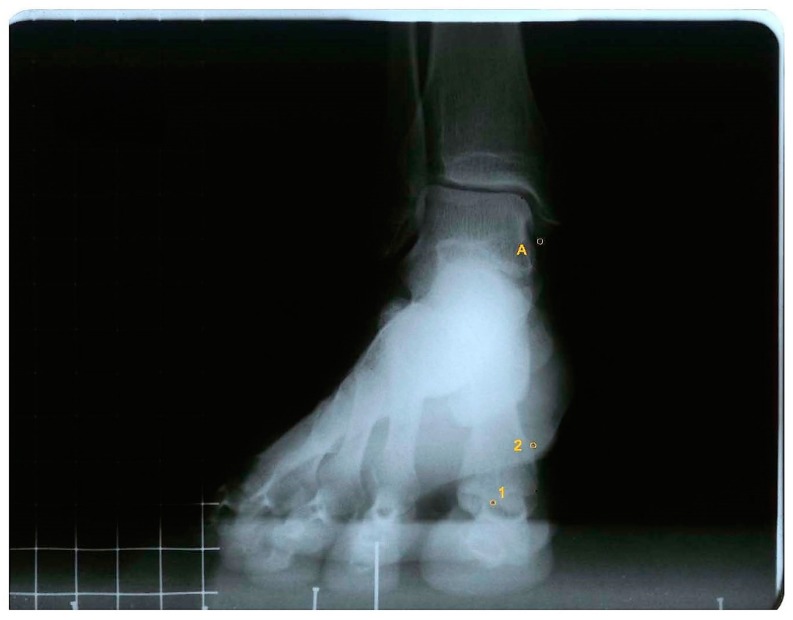
Antero-posterior radiograph of a participant with the first ray in a neutral position, showing point A (most distal point of the tibial malleolus), point 1 (intersesamoid crest), and point 2 (superomedial tubercle of the head of the first metatarsal).

For measurements, the AutoCAD^®^ software was used (AutoCAD 2016; Autodesk Inc., San Rafael, CA, USA). One point was drawn in the intersesamoid crest (point 1) and one in the superomedial tubercle where the medial collateral ligament of the first metatarsophalangeal joint begins (point 2). A third point was marked in the most inferior tip of the tibial malleolus (point A), which acted as a fixed point (Figure 1).

**Figure 2:**
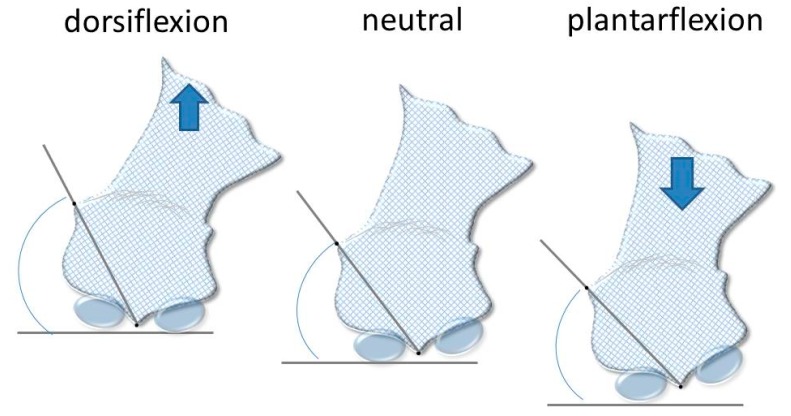
Model representing the angle used to measure rotation in the frontal plane.

**Figure 3:**
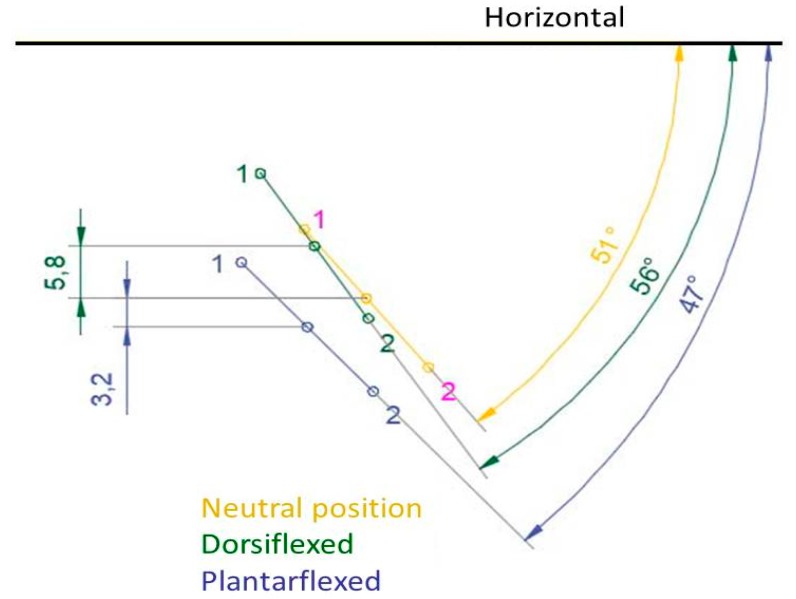
Example of measurement of the frontal plane rotation with AutoCAD in one participant.

To obtain the first ray displacement in the sagittal plane the distance was measured in millimeters from point A to point 1, in the three positions, plantarflexed, neutral, and dorsalflexed. To measure the movement in the frontal plane, two lines were drawn to find the angle that formed between them: one line between points 1 and 2, and the other being a horizontal line, parallel to the ground (Figure 2 and Figure 3).

To mathematically determine the whole ROM of the first ray in the two planes, the curve that the first metatarsal described was studied without the imposition of continuity established in the statistical measurement and based on a mathematical fit. The points that generated the final fit were established and defined by their coordinates X and Y (rectangular coordinates in a reference system based on the affine space). *Point 1* was used as the mobile point of the three radiographs of each participant with respect to the fixed point A.

*Point A* was also established as the origin of the coordinates of the system, obtaining a value X and a value Y, resulting from the measurements that were made in the direction of the horizontal axis X and the vertical axis Y of *point 1* in each radiograph. Three coordinate points were thus obtained (X,Y), defining the position of *point 1* with respect to *point A* in the plantarflexed, neutral, and dorsalflexed positions. Hence, a function could be generated which described a curve that fulfilled the condition of passing through these points. The curve described was determined by carrying out the operation of taking points in the three radiographs of each participant.

### 2.4. Data Analysis

The statistical analysis of the data was carried out via the SPSS Statistics^®^ software, version 22 (IBM, Corp, Armonk, NY, USA) for Windows^®^.

It was noted if the displacement of the first ray was different between the left and right feet, or between men and women. The Shapiro-Wilks test was used to determine if the data followed a normal distribution, and the Mann-Whitney U test and the T Student test for independent samples were employed to carry out the comparisons. All of the values of *p* < 0.05 were considered to be statistically significant.

To check the intra-rater reliability of this measurement procedure, ten radiographs were chosen at random, and the distance from point A to point 1, in the three positions, as well as the angle formed by one line between points 1 and 2, and the horizontal, were measured twice, one month separating each measurement. The intraclass correlation coefficient (3,1) was calculated using the data obtained from these measurements.

The MatLab R2017a software (The Mathworks, Natick, MA, USA) was used to find a mathematical function which described the first ray curve of movement. The neutral position of the first ray was established as a common point between all of the participants to generate a coherent relation between the images of them, and to carry out a general study that could be applicable to all of them. Thus, all of the points obtained in the same reference system were superposed. The measurement existing between the neutral and the plantarflexed positions was taken, obtaining three values (X,Y) in the horizontal direction and vertical direction, respectively, the value (0.0) being the reference of the neutral position. In the same way, the values between the neutral position and the dorsalflexed position were obtained. Consequently, two points of coordinates appeared for each participant (X,Y), referring to the plantarflexed and dorsalflexed positions. A total of 72 points with distribution (X,Y) were established to seek the mathematical equation that best fitted the cloud of points that were obtained. The fit was done via a polynomial function that was determined with the least squares method.

## 3. Results

Although the initial sample for this study consisted of 40 participants, two different examiners could only clearly identify the points to mark in 36 radiographs. Thus, the final sample was 36 participants (*N* = 36), 23 women and 13 men, with an average age of 25.42 ± 3.97 years old (range 21–37) and a BMI of 23.85 ± 2.93 kg/m^2^. 17 right feet and 19 left feet were included. The lowest value of ICC for the six measurements (sagittal plane linear displacement and angular rotation in the frontal plane, both in the three positions) was 0.952. This suggests that the reproducibility of the measurements was good.

Table 1 shows the dorsal, plantar, and total displacement of the first ray in the sagittal plane. The sagittal plane results are reported as linear displacements (mm). The dorsal displacement was the difference found between distance A-1 with the first ray in the neutral position, and this same distance with the first ray maximally dorsiflexed. The plantar displacement was the distance found between distance A-1 with the first ray in the neutral position, and this same distance with the first ray maximally plantarflexed. The total displacement was the difference found between distance A-1 with the first ray being maximally dorsiflexed and maximally plantarflexed (the sum of the dorsal displacement and the plantar displacement). Significant differences were not found in these three variables between men and women (dorsal *t* = 0.81, *p* = 0.425; plantar *t* = −1.62, *p* = 0.115; total *t* = −0.49, *p* = 0.630), or between left feet and right feet (dorsal *t* = 0.50, *p* = 0.619; plantar *U* = 142.00, *p* = 0.537; total *t* = 0.97, *p* = 0.340).

Table 2 shows the results of the FRM in the frontal plane. The frontal plane results are reported as angular displacements (degrees). The degrees of inversion were obtained in calculating the difference between the aforementioned angle with the first ray in a neutral position and in maximum dorsiflexion. The degrees of eversion were obtained calculating the difference between this angle with the first ray in a neutral position and in maximum plantarflexion. Significant differences were not found in these variables between men and women (angle in neutral position *t* = −0.05, *p* = 0.962; angle in maximum dorsalflexion *t* = 0.25, *p* = 0.801; angle in maximum plantarflexion *t* = −0.42, *p* = 0.677; inversion *t* = 0.51, *p* = 0.614; eversion *t* = 0.81, *p* = 0.425), or between left feet and right feet (angle in neutral position *t* = 0.91, *p* = 0.368; angle in maximum dorsalflexion *t* = 0.37, *p* = 0.724; angle in maximum plantarflexion *t* = 0.60, *p* = 0.551; inversion *U* = 138.00, *p* = 0.451; eversion *t* = 0.79, *p* = 0.435).

**Figure 4:**
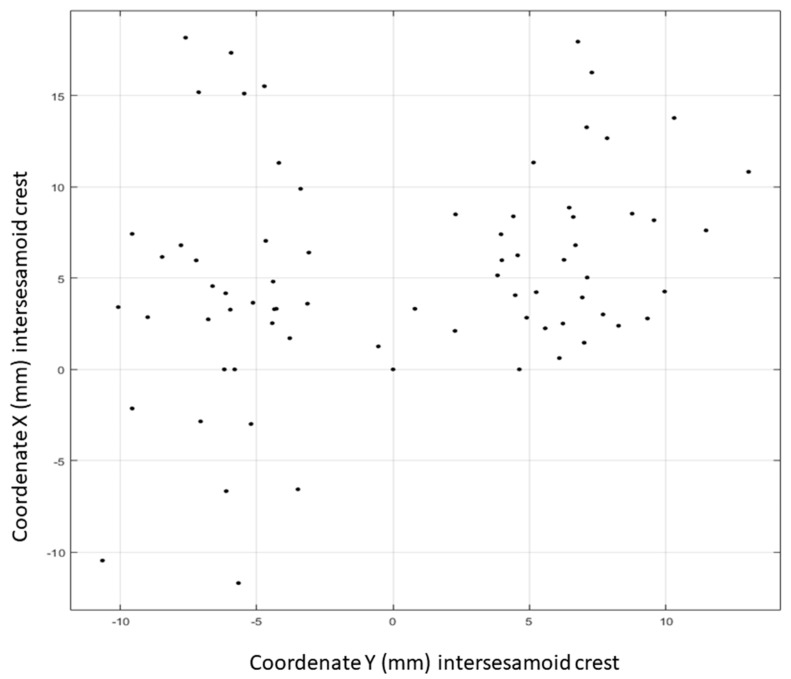
Spatial distribution in rectangular coordinates of the position of the mobile point 1 (intersesamoid crest) with the first ray in maximum plantarflexion and maximum dorsalflexion.

**Figure 5:**
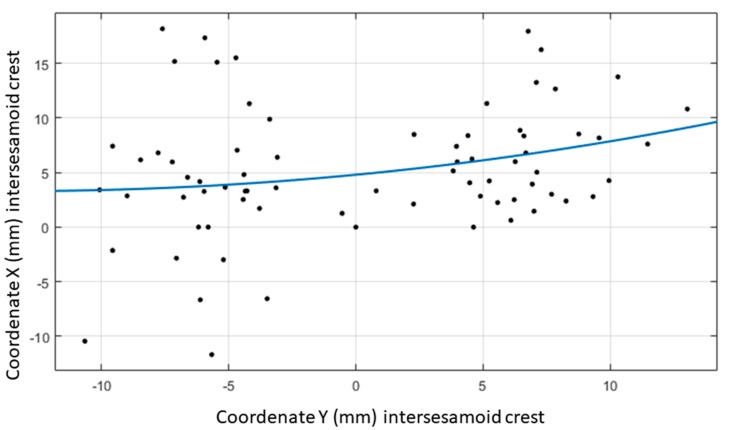
Quadratic function (parabolic curve) having the best fit with the cloud of points obtained via the mathematical calculation, represented within the zone of influence of the data imposed to determine it.

A cloud of points was generated (Figure 4) using the 73 points that were established with distribution (X,Y). The polynomic function which best fitted this cloud of points was obtained via the least squares method. This proved to be a second-degree equation of the type: $f\left(x\right)=ax^{2}+bx+c$. The quadratic function represented was the equation of a symmetric parabola with respect to a vertical axis. Studying the distribution of the points, this possible symmetry had to be established with respect to the horizontal axis, so the equation was defined as: $f\left(y\right)=ay^{2}+by+c$. The curve that best adapted according to a grade two polynomic function had the values a, b and c as coefficients. Points were introduced into the software interchanging coordinate X for coordinate Y to be able to generate that curve with a symmetric tendency with respect to the horizontal axis. In this way, the horizontal axis of the radiographs appeared as the vertical axis of the image obtained from the software. Introducing the 73 points denominated (YP1D, XP1D), the equation obtained was: $f\left(y\right)=0.0083y^{2}+0.2230y+4.7983$ (Figure 5).

## 4. Discussion

The aim of this research was to quantify the normal static ROM of the first ray. As far as we know, this is the first study that quantifies the inversion and eversion of the first ray together with dorsiflexion and plantarflexion via radiograph images in subjects with a normal first ray. The modified Coleman method of tacking displacement (static range of motion) of the first ray is well accepted in the literature. However, the method that was used in this study to quantify measuring motion from points digitized on radiographs as well as the math computation for tracking displacement, are novel. The measurements introduced in this work allow for quantifying both the linear displacement in the sagittal plane and the angular displacement in the frontal plane. This could be considered an advantage when compared to how other works in the literature have used the modified Coleman test in lateral radiographs to objectify the measure of first ray motion.

To the best of our knowledge, the first author who studied the movement of the first ray was Hicks in 1953 [13]. He noted that the first ray made dorsalflexion-inversion and plantarflexion-eversion movements, having a total ROM of 22 degrees. Kelikian stated that the first TMTJ allows 10–15 degrees of passive movement in the sagittal plane [23]. Ebisui determined that the first ray made dorsiflexion-inversion movements during the pronation of the foot and plantarflexion-eversion movements during supination and that these movements occurred around the axis that was described by Hicks [24]. This finding is also shared by Sarrafian [25], and Root et al. [14], who sustain that for each degree of displacement in the sagittal plane another degree of movement takes place in the frontal plane, although these authors do not report the studies from which they have drawn this conclusion. Although what is most widespread and accepted is that dorsalflexion is accompanied by inversion and plantarflexion by eversion, there are authors who uphold that during subtalar pronation, the dorsalflexion of the first ray is accompanied by eversion [26,27,28,29], although these studies include participants with hallux abducto-valgus, not with normal first rays. A possible explanation would be that when the first ray reaches its maximum capacity of dorsalflexion and inversion, and the rearfoot continues pronating, the movement produced in all of the foot (and therefore also in the first ray) is an eversion movement. We also think that in hallux abducto-valgus, due to the metatarsus primus varus deformity, the excessive tension of the soft tissues laterally inserted in the first metatarsophalangeal joint (lateral head of the flexor hallucis brevis, adductor hallucis) could produce this eversion of the first metatarsal during the dorsalflexion. In all of the participants included in this study, except in four cases, the angle used to measure the first ray rotation was greater in the maximum dorsalflexion of the first ray than in the neutral position and was lesser in the maximum plantarflexion than in the neutral position. This means that inversion took place when the head of the first metatarsal was moved upward and eversion occurred when it was moved downward.

We have only found one previous study that measures the displacement of the first ray in millimeters for the sagittal plane and degrees for the frontal plane [4]. The results obtained in vitro were that the total ROM of the first ray in the sagittal plane was 12.4 (SD 3.4) mm and was 8.2 (SD 4.1) degrees in the frontal plane. Our values were similar in the sagittal plane. However, in the frontal plane the total movement of the first ray was three degrees less. This may be due to amputating the hallux and sectioning the soft tissues around it causing the first ray to have a greater freedom of movement in the frontal plane than that which could be observed in vivo. On the other hand, Kelso et al. determined that 0.77 degrees of movement took place in the frontal plane for each millimeter of displacement in the sagittal plane [4]. To compare this figure with our results, the degrees of rotation in the frontal plane (5.66) were divided between the millimeters of displacement in the sagittal plane (12.59). The result was that for each millimeter of displacement in the sagittal plane the first ray made a rotation of 0.45 degrees in the frontal plane.

Many methods have been proposed to measure FRM and one of the most common is to manually move the first metatarsal in the dorsal and plantar directions with one hand while stabilizing the lesser metatarsals with the other. Although this technique can be used in a clinical setting without special equipment, its validity and reliability is questionable [30,31]. Based on clinical experience, Root et al. proposed that the normal movement of the first ray was 5 mm of dorsiflexion and 5 mm of plantarflexion in the sagittal plane [22], but they did not directly measure FRM with any scientific means. Later, various studies have been carried out using different measurement instruments to try to quantify FRM [9,10,32]. However, none of those instruments takes into account the first ray movement in the frontal plane.

Klaue et al. reported that the dorsiflexion of the first ray was 5.3 mm [10]. Various authors have then used this device, obtaining diverse results: 4.4 mm [33], 4.9 mm [34], 5.6 mm [35], 6.85 mm [36], and 7.2 mm [37]. Glasoe et al. observed that the first ray dorsiflexion was 4.2 mm [9]. Later, Cornwall et al. [8,38], used this device and obtained 6.2 mm [38] and 6.6 mm [8]. FRM in dorsiflexion is commonly and importantly measured in the literature. Dorsiflexion ranges from 3 to 8 mm in healthy adults and one article authored in 2006 by Glasoe and Coughlin (who in a long series of independent and unaffiliated research studies measured with two different types of mechanical devices) made a consensus statement that FRM averages 5 mm in healthy adults, and that values exceeding 8 mm indicate hypermobility [39]. The measure of 6.47 mm in dorsiflexion reported in the present study equates FRM dorsiflexion values that were reported in the literature [8,36,37]. Most of this data was recorded by researchers who used singularly designed and well tested mechanical devices (for reliability and validity) for this purpose. Since these dorsiflexion results match with what has been reported in the literature (significant differences not being found between men and women, or between left feet and right feet, thus not influenced by gender or by the side), it might be considered as an indirect way of validating the results reported.

With respect to the mathematical equation obtained, this defines the curve that best fitted the points described. By knowing one point of the coordinate axes (i.e., millimeters of dorsal displacement), another point could be obtained. In this way, FRM could be represented in a coordinates system (X,Y) as a curve. The curve that was obtained in the study fulfills the premises of continuity and fits the cloud of points, fundamental in the function’s mathematical development. Along with these two criteria, a polynomic function was taken as a calculation basis as it is one of the most usual solutions for the fit of curves from the engineering point of view [40], especially if the method of obtaining it is via a fit. These findings might be useful in guiding the future design of instruments for clinically quantifying first ray mobility.

This study has certain limitations. For example, the methodology used did not take into account the anatomic orientation of the first TMTJ, as two-dimensional images have been used to value three-dimensional elements. It must be clarified that this is not an attempt to impute three-dimensional motion from radiographs taken in a single plane, but means to show whether the first ray made a rotational motion when moved upward and downward. In addition, we have tried to decrease the risk of errors by following a standardized and rigorous X-ray protocol. In previous investigations it has been demonstrated that when the radiographs are done with the same protocol, the differences with reality can be not significant, at least regarding the first metatarsal-digital segment [41]. Another limitation is that a normal range of motion was not differentiated according to the corresponding joint levels of the medial foot column. Instead, the authors have tried to quantify the first metatarsal head mobility as it is clinically relevant for the manual evaluation of the first ray. Finally, it must be taken into account that only normal first rays have been included, so the results may vary with other conditions (i.e., hallux abducto-valgus or hallux rigidus). More research is necessary to study the mobility of the first ray in these deformities.

## 5. Conclusions

It was noted that the head of the first metatarsal inverted when carrying out dorsiflexion in the sagittal plane and everted when doing plantarflexion. Hence, movements in both the sagittal and frontal planes were produced. The values that were obtained in the sagittal plane were: dorsalflexion 6.47 (SD 2.59) mm, plantarflexion 6.12 (SD 2.55) mm, and total movement 12.59 (SD 3.92) mm. The movement in the frontal plane was 2.69 (SD 4.03) degrees of inversion during the dorsalflexion, and 2.97 (SD 2.72) degrees during the plantarflexion. 0.45 degrees of movement in the frontal plane took place in the participants of this study for each millimeter of displacement in the sagittal plane.

The equation of a symmetric parabola was calculated with respect to a vertical axis that best represented the FRM in the participants in this study. Studying the distribution of the points that were obtained in the images of the study, the equation that best represented the curve of movement was $\left(y\right)=0.0083y^{2}+0.2230y+4.7983$. These findings might be useful in guiding the future design of instruments to clinically quantify first ray mobility.

## Figures and Tables

**Table 1:** Displacement of the first ray in the sagittal plane in all the participants (mean ± standard deviation), and in men and women separately. The displacements have been obtained calculating the distance between point A and point 1 with the first ray in a neutral position, in maximum dorsalflexion, and in maximum plantarflexion.

	Total Sample (*N* = 36)	Men (*N* = 13)	Women (*N* = 23)
Dorsal Displacement (mm)	6.47 ± 2.59	6.94 ± 3.34	6.20 ± 2.09
Plantar Displacement (mm)	6.12 ± 2.55	5.22 ± 2.21	6.62 ± 2.63
Total Displacement (mm)	12.59 ± 3.92	12.16 ± 4.83	12.83 ± 3.41

**Table 2:** Movement of the first ray in the frontal plane in all of the participants (mean ± standard deviation), and in men and women separately. The movements have been obtained calculating the difference between the angle formed by a horizontal line and a line that connects points 1 and 2, with the first ray in a neutral position, in maximum dorsalflexion, and in maximum plantarflexion.

	Total Sample (*N* = 36)	Men (*N* = 13)	Women (*N* = 23)
Angle in maximum dorsalflexion (degrees)	55.91 ± 6.86	56.31 ± 6.81	55.70 ± 7.03
Rotation during the dorsalflexion (degrees)	2.69 ± 4.03	3.15 ± 3.62	2.43 ± 4.29
Angle in neutral position (degrees)	53.22 ± 6.28	53.15 ± 6.99	53.26 ± 6.02
Angle in maximum plantarflexion (degrees)	50.25 ± 5.91	49.69 ± 6.80	50.56 ± 5.48
Rotation during the plantarflexion (degrees)	2.97 ± 2.72	3.46 ± 2.76	2.70 ± 2.72
Total rotation	5.66 ± 4.78	6.61 ± 3.92	5.13 ± 5.22
